# Chronic Traumatic Encephalopathy: The Impact on Athletes

**DOI:** 10.7759/cureus.532

**Published:** 2016-03-14

**Authors:** Michael A Galgano, Robert Cantu, Lawrence S. Chin

**Affiliations:** 1 Neurosurgery, SUNY Upstate Medical University; 2 Neurosurgery, Emerson Hospital

**Keywords:** chronic traumatic encephalopathy, mild traumatic brain injury, repetitive traumatic brain injury, athletics, sports, high impact sports

## Abstract

Chronic traumatic encephalopathy (CTE) is a devastating neuropsychological condition afflicting a small percentage of athletes partaking in high-impact sports. The onset of symptoms lags years behind the inciting events. Repetitive minor head injuries are felt to be the main etiology behind CTE. Routine radiographic imaging generally is unremarkable in cases of CTE. Functional magnetic resonance imaging (fMRI), magnetic resonance spectroscopy (MRS), and diffusion tensor imaging (DTI) are advanced MRI-based sequences that have shown promise in detecting early radiographic findings that may be reflective of CTE. Progressive neuronal loss is the histopathological hallmark of this neurodegenerative disease. Strategizing earlier detection techniques is paramount in delivering optimal care to athletes afflicted with CTE.

## Introduction and background

### Epidemiology

One and a half million Americans suffer traumatic brain injuries without loss of consciousness or hospitalization on an annual basis [1]. Most of these injuries are defined as "concussions". They account for 10% of all head and spinal cord injuries, and the Center for Disease Control attributed 207,830 emergency room visits per year between 2001 and 2005 to non-fatal sports-related head injuries [2]. A study of high school athletes found that all sports carried the risk for concussions with boys’ football being the highest at 63% of the total, followed by wrestling (10%), soccer (6%), with the remainder in basketball, softball, baseball, field hockey, and volleyball [3-4]. A sport that also poses a very high risk of chronic traumatic brain injury not seen in high school athletics is professional boxing. In a previous well-designed prevalence study conducted four decades ago, it was estimated that approximately 17% of retired professional boxers exhibit characteristics of chronic traumatic brain injury [5].

A concussion is generally defined as a mild injury to the brain. The predominating etiology is often caused by traumatic biomechanical forces. Radiographic evidence of damage with routine techniques, such as CT and MRI, is uncommon. A direct blow to the head may cause the concussive event. Acceleration forces applied to the head without direct trauma can also be a cause. A concussion does not imply the loss of consciousness and 90% of concussions due to sports present in such a way [6]. Headaches, dizziness, confusion, disorientation, and blurred vision are common symptoms of concussions.

Contrary to previous beliefs, concussions are not benign. There is mounting evidence showing that concussions or mild traumatic brain injury (mTBI) have more serious complications, particularly when they occur repeatedly. Sports-related chronic traumatic encephalopathy (CTE) manifests as a progressive worsening of cerebral neurological symptoms, initiated by, and perhaps worsened by, repetitive concussions and subconcussive injuries. While the precise incidence and prevalence of CTE is unknown, recent epidemiological data have shown that 17% of the individuals with repetitive mTBI may develop CTE; however, the severity and the frequency of repetitive injury needed to cause CTE continues to be elusive [7-10].

Research regarding concussions in the youth population remains rather limited, despite an increasing number of reported individuals being treated in US emergency departments. According to a report from the Committee on Sports Concussions in Youth, individuals aged 19 and under who were seen in an emergency department for concussions and other non-fatal sports and recreational-related TBI’s increased from 150,000 in 2001 to 250,000 in 2009 [11]. Because of this increased awareness, there are new protective devices being advertised for young athletes, and some of these are sold under the premise that they may limit the concussion risk. However, there continues to be no solid scientific evidence supporting these devices. It is known that the youth population is rather resistant to reporting concussive events. This attitude, unfortunately, perpetuates further concussive events, since a history of concussions increases the risk of further concussions. Athletes who return to play before their first concussive event is healed place themselves at a significant risk for a second significant brain injury and the adverse effects of the cumulative process.

### History

In 1928, Martland introduced the term "punch drunk" to describe neurological symptoms, such as confusion, tremors, slowed speech, and the gait disturbances, seen in boxers suffering from repeated blows to the head [11]. This came to be known as "dementia pugilistica" by Millspaugh [12], and then Courville introduced the term, “psychopathic deterioration of pugilists” [13]. The neuropathology of CTE was first described by Brandenburg and Hallevorden and later by Corsellis who found several characteristic areas of damage: septum pellucidum, adjacent periventricular gray, frontal, and temporal lobes, substantia nigra, cerebellar scarring, and diffuse neuronal loss [7, 14-15]. Although originally described in boxers, CTE has since been found in other sports, including football, wrestling, and hockey, as well as in individuals suffering repetitive brain trauma who were not athletes [7, 14]. More recently, CTE has been found in military blast victims.

## Review

### Clinical presentation

Patients with CTE present with headaches, dizziness, an unsteady gait, fatigue, and dysarthria. In addition, they may have cognitive and psychosocial symptoms, such as memory loss, attention deficit, difficulty in concentration, slow information processing, confusion, loss of judgment, irritability, emotional distress, and show an inability to stay employed. In severe cases, there is a progressive slowing of movement, a propulsive gait, tremor, masked facies, deafness, dysarthria, dysphagia, ptosis, and other ocular abnormalities [7]. Clinical deterioration in CTE typically occurs in three stages [7, 14]:

Stage 1 - Affective disturbances and psychotic symptoms,

Stage 2 - Social instability, erratic behavior, memory loss, and initial symptoms and signs of Parkinsonism,

Stage 3 - General cognitive dysfunction, the progression of dementia, speech, and gait abnormalities, or full-blown Parkinsonism.

The initial symptoms are due to damage to the limbic system resulting in behavioral symptoms, such as emotional lability, aggression, and violence. Hippocampal as well as the medial thalamus and entorhinal cortex involvement account for the memory disturbances. Disinhibition occurs as more frontal lobe involvement develops, and degeneration in the temporoparietal and occipital cortex results in visuospatial symptoms. Finally, in the late stages, degeneration of the substantia nigra causes Parkinsonian symptoms [7, 16-17].

The diagnosis of the CTE related to sports is based on a history of repetitive concussions, which may have been subclinical, evidence of disease progression, and the neurological exam. CT and MRI scans can be helpful for excluding other causes of neurological symptoms, such as chronic subdural hematoma or brain tumor. Imaging may also identify nonspecific changes, such as atrophy, encephalomalacia, and demyelination.

### Notable diagnoses in athletes

American football has felt the greatest impact of CTE amongst all other sports. In 2005, Omalu, et al. identified pathology consistent with CTE in a National Football League (NFL) player who had died of atherosclerotic heart disease 12 years after a long career in the NFL [18]. Interestingly, family members noticed that he had suffered from memory deficits and Parkinsonian symptoms.

More recent cases have resulted in greater media attention. Four years after competing in the Superbowl, NFL star Junior Seau committed suicide in 2012. This sparked significant awareness after the family of the NFL player decided to sue the National Football League. Most notably, the 1987 NFL Man of the Year Award winner, Dave Duerson, committed suicide in early 2011. The 50-year-old former Chicago Bears safety, who died of a self-inflicted gunshot wound to the chest, left a note asking that his brain be “given to the NFL’s brain bank” at the Boston University Center for the Study of Traumatic Encephalopathy (BU CSTE). Through his work with the football players union, Duerson was aware of CTE and other cases of retired players who had suffered from CTE-like symptoms. For example, Mike Webster, Justin Strzelczyk, and Terry Long, all of the Pittsburgh Steelers, Andre Waters of the Philadelphia Eagles, Chris Henry of the Cincinnati Bengals, John Grimsley of the Houston Oilers, and Tom McHale of the Tampa Bay Buccaneers, all died after years of strange, erratic, and sometimes overly aggressive behavior.

Omalu, et al.* *reported a case of chronic traumatic encephalopathy in an American professional wrestler, Chris Benoit. He had sustained multiple blows to the head over his career. Initially, he presented with significant depression at the age of 36, and eventually committed a murder-suicide involving his wife and son a few years later. An autopsy later revealed randomly distributed neurofibrillary and ghost tangles in the neocortex, subcortical ganglia, and brainstem nuclei [19-20].

Ryan Freel was the first major league baseball player to be diagnosed with chronic traumatic encephalopathy. He died of a self-inflicted gunshot wound in December 2012. A report of Freel’s condition was presented from the Boston University Center for the Study of Chronic Traumatic Encephalopathy and Sports Legacy Institute. It stated that he suffered from Stage II CTE when he died. Freel suffered multiple concussions during his playing career and was hospitalized in 2007 after colliding with another player.

Other notable cases from BU CSTE included Owen Thomas, a 21-year-old football player at the University of Pennsylvania who committed suicide by hanging himself and was subsequently diagnosed with CTE. With more than a million teenagers competing in football every year, it is clear that the risk of CTE looms as a potential public health disaster.

### Pathological findings

Brain specimens with CTE show a reduction in weight, enlargement of the ventricular system, especially the lateral and third ventricles, thinning of the corpus callosum, atrophy of the cerebral hemispheres, mesial temporal lobes, thalamus, mammillary bodies, olfactory bulbs, and brainstem, pallor in the substantia nigra and locus coeruleus, a cavum septum pellucidum, and scarring of the cerebellar tonsils.

Neuronal loss and gliosis are the most common microscopic findings in CTE and are usually associated with neurofibrillary degeneration, especially in the hippocampus, subiculum, entorhinal cortex, amygdala, subcallosal and insular cortex, frontal and temporal cortex, mammillary bodies, substantia nigra, locus coeruleus, nucleus accumbens, medial thalamus, pars compacta, and pars reticulata [7, 21-23]. The pathognomonic neuropathological signs of CTE are finding tau-immunoreactive neurofibrillary tangles (NFTs) with preferential involvement of superficial cortical layers, an irregular and patchy distribution in the frontal and temporal cortex, a propensity for sulcal depths, and a perivascular, periventricular, and subpial distribution. Unlike Alzheimer’s disease (AD), which shares a similar neuropathology, tau deposition in CTE is denser and preferentially found in neocortical layers II and III. Beta-amyloid deposition in CTE is also more sporadic compared to AD [7].

### Pathophysiology

Chronic traumatic encephalopathy likely results from progressive neuronal loss, but the exact mechanisms are unclear. CTE caused by repetitive mTBI continues to progress decades after the injuries have stopped, indicating that once the cascades are initiated, they continue to execute their effects and that the longer the individual lives, the worse the symptoms become [7, 16]. The severity threshold of the brain injury that can initiate these progressive chronic neurodegenerative changes, as well as the frequency, are still unknown.

The patchy cortical distribution of NFTs suggests that their location is correlated with areas of direct mechanical trauma from blows to the side or top of the head. An ischemic basis of injury comes from the observation that tau deposition tends to be in the depths of the cortical sulci [7]. The associated breakdown of the blood-brain barrier following brain injury with the release of local neurotoxins potentially explains the perivascular clustering of NFTs [24]. Buee, et al. examined the microvasculature in patients with dementia pugilistica and found decreased microvasculature density and tortuosity, which corresponds to the laminar distribution of tau-positive NFTs [25]. In addition to tau, other protein inclusions may play an important role in CTE. The DNA binding protein TDP-43, along with tau, has been observed in all of our brains with CTE and the brains and spinal cords of athletes presenting simultaneously with CTE and motor neuron disease, suggesting a common link between CTE and sporadic amyotrophic lateral sclerosis [26]. While the exact function of TDP-43 is unknown, its overexpression causes neuronal degeneration and cell death in animal models [27]. One possible hypothesis is that mild traumatic brain injury causes axonal shear with cytoskeletal disruption.

As part of the injury response, TDP-43 is upregulated and binds to neurofilament mRNA in order to stabilize the transcript. Because this protein is prone to aggregation, pathological TDP-43 deposits form, resulting in neurotoxicity and cell death.

### Symptomatic management

Athletes engaging in high impact sports, whether it is at the professional, collegiate, or high school level, should be counseled on the importance of reporting symptoms as well as “occult” concussive events not visibly recognized by team trainers. If an individual is known to have sustained repetitive concussive or subconcussive blows to the head over time, they should be referred to a specialty center, such as a concussion clinic. Such clinics provide individuals with a multidisciplinary approach to their care, whereby psychiatry, neurology, and neurosurgery services can provide care as needed. It is important for ‘at-risk’ athletes to have surveillance for suicidal tendencies, access to behavioral/counseling services, and routine neurocognitive, neuropsychiatric, and functional capacity evaluations.

### Prevention

Because there are no known treatments for CTE, prevention of concussion and subconcussive blows is the only option. Prevention strategies must be sport-specific. For example, several risk factors for CTE in the sport of boxing have been identified. Studies have shown that older boxers, those with long careers, poor performers, and individuals who have suffered several concussive blows are at the highest risk. These athletes should be identified and be required to go undergo a more extensive neurological evaluation. A more controversial but interesting aspect of prevention is genetic testing. Apo E4 is speculated to be a susceptibility gene in both football and boxing. The presence or absence of this gene may, in fact, be an important modifier of a particular athlete's overall risk of developing CTE [5].

An important part of prevention is the improved recognition of concussion on the playing field. It has been shown that suffering the first concussion increases the likelihood of a second concussion up to threefold [28]. Standardized concussion tools have been developed that assist physicians and trainers in this assessment. Concussion grading scales, such as the Cantu system, use the loss of consciousness, post-traumatic amnesia length, and post-concussion signs and symptoms to determine severity (Table 1).

**Table 1:** Cantu Evidence-Based Grading System for Concussion *LOC—Loss of consciousness; ** PTA – Post-traumatic amnesia; *** PCSS – Post-concussion signs and symptoms

Grade	Level of Consciousness
Grade 1 (mild)	No LOC*, PTA** < 30 min, PCSS *** < 24 h
Grade 2 (moderate)	LOC < 1 min or PTA ≥ 30 min <24 h or PCSS ≥ 24 h < 7 d
Grade 3 (severe)	LOC ≥ 1 min or PTA ≥ 24 h or PCSS ≥ 7 d

Return to play (RTP) guidelines have been developed that will hopefully decrease the risk of catastrophic injuries, such as the second impact syndrome as well as CTE. The RTP guidelines are based on concussion severity and require that the athlete is symptom-free at rest and exertion for at least one week before being considered ready to return to the sport (Table 2).

**Table 2:** Cantu Return to Play Guidelines Note: Asymptomatic athlete is free of symptoms for one week

Grade	1st Concussion	2nd Concussion	3rd Concussion
1	When asymptomatic, one week	In two weeks if asymptomatic	Terminate season; may return next season if asymptomatic
2	When asymptomatic, one week	In one month if asymptomatic	Terminate season; may return next season if asymptomatic
3	In one month if asymptomatic	Terminate season; may return next season if asymptomatic	Terminate season; may return next season if asymptomatic

When a second concussion is suffered, the time period that the athlete must wait varies from two to four weeks, depending on the concussion severity. After three concussions (two, if severe), the player should terminate. There has been tremendous research and development in designing new helmets, but their effectiveness in preventing concussions has not been proven. Mouth guards can reduce dental injuries but also have little impact on preventing concussions. Padded goal posts in soccer can help prevent more serious brain injuries in this sport. In addition, lighter waterproof balls can be considered to reduce the impact of the ball striking the head during heading. However, there is some concern that protective equipment results in play that is more aggressive and may paradoxically increase the incidence of injuries [29].

### Future direction and research

Advanced Imaging

It is widely accepted that concussion results mainly in functional disturbances as opposed to gross structural damage [30]. In concussed athletes, routine neuroimaging findings are generally negative. However, more novel imaging techniques using MRI, such as diffusion tensor imaging (DTI) and functional MRI (fMRI), may result in an earlier diagnosis of concussion. Concussive impacts on the brain often result from acceleration/deceleration forces. The biomechanical strain on the white matter may, in fact, lead to alterations in brain metabolism and electrophysiology. In 1967, Peerless and Rewcastle specified “shear-strain injury" or “traumatic axonal injury". This particular type of injury to the axons is generally seen at the junction between the gray and white matter. The shearing that takes place at this junction from acceleration/deceleration forces targets this area of the brain because of the physical make-up of the two different tissue types at this junction. The gray and white matter differs with respect to their rigidity. One of the main properties contributing the tissue characteristics of white matter is the myelin sheath which surrounds the axons [31]. Hovda and colleagues have published recent results that indicate automated analysis of MRI scans can provide clinicians with information that may help in assessing patients with mTBI. Zhang, et al. compared MRI with DTI in 49 professional boxers and found only nonspecific white matter changes on MRI, but diffusion anisotropy analysis revealed a decrease in the average diffusion constant and whole brain diffusion [28]. These changes may indicate an early response to a concussion in athletes and could become a useful tool for both detecting early damage and monitoring long-term neurological deficits.

Diffusion tensor imaging (DTI) is an MRI-based imaging modality, which provides information on the microstructural integrity of the brain white matter tracts. It does this by measurement of water molecule diffusion within the white matter. Because DTI is sensitive to white matter tracts, it has the ability to give us radiographic information regarding their disruption [31]. Some studies have shown a correlation between fractional anisotropy, which is a measure of axonal integrity, and functional tests of post-concussive states, such as eye tracking [32]. DTI can also provide information in regards to gray matter integrity as well. Gliosis and necrosis found on a DTI scan of an athlete may, in fact, be an indicator of brain injury from repetitive concussions [33].

Functional magnetic resonance imaging (fMRI) is another imaging modality that has the ability to capture neuronal activation via activation changes in blood-oxygen-level-dependent (BOLD) analysis. It is presumed that the neural substrates of a particular task are represented by changes in signal intensity. The changes in signal are due to changes in the state of oxygenation of hemoglobin in the specific areas of the brain, which are activated during these tasks. Studies related to fMRI have a focus on working memory, as this is what has been primarily examined in regards to this imaging technique. Some may conclude that sports-related concussions are primarily a disorder of frontal network functioning [33].

Magnetic resonance spectroscopy (MRS) is another non-invasive imaging technique that has the ability measure neurometabolites. It does this via proton magnetic resonance spectroscopy. Changes in NAA, choline, and Cr ratios demonstrated by MRS have been associated with individuals who have experienced a traumatic brain injury. In one particular study by Vagnozzi, it was shown that athletes reported resolution of post-concussive symptoms by post-injury day 3, despite continued MRS abnormalities [34]. Further findings also suggest that complete metabolic recovery may be delayed if a second concussion occurs within a short time interval from the first and that further abnormal metabolic changes may take place. This suggests that there are chronological differences in clinical recovery and metabolic recovery following a mild traumatic brain injury [33].

Small, et al.* *more recently has looked at PET scanning of brain tau protein in retired NFL players. Preliminary data showed that brain tau protein markers in all subcortical regions, as well as the amygdala, were higher in retired NFL players than controls. There were a rather small sample size and lack of autopsy confirmation in regards to this small study, but this does show some potential for offering premorbid identification of neurodegeneration in athletes [35].

Earlier Diagnosis of CTE

The only true way to diagnose CTE is post-mortem via an autopsy. Recent work by Maruyama and colleagues has been promising in the field of *in vivo *radiographic detection of tau inclusions. This group has developed a class of tau ligands specifically utilized for the visualization of diverse tau inclusions within the brains of individuals afflicted with tauopathies, such as Alzheimer’s disease. These ligands are able to penetrate the blood-brain-barrier and have the capability of binding to and ultimately visualizing aggregations of intracellular tau protein. The group’s *in vivo *imaging studies have demonstrated intensified signals in tau-rich regions of the brain [36]. Once such earlier methods of tau aggregate detection are further refined, the possibility of diagnosing CTE prior to its deleterious effects taking place on the individual may be possible. Clinicians will then have a more definitive diagnosis for which an adequate treatment plan can be formulated.

Animal Models of CTE

A multitude of traumatic brain injury animal models has been created over the years, but no model has sought to replicate the spectrum of neurobehavioral changes seen in CTE until recently. Huang and colleagues have developed a hybrid animal model combining two of the more popular TBI models, in an effort to demonstrate neurobehavioral changes concurrent with CTE. Their closed-head injury model is unique in that it includes unanesthetizedmice as the subjects. Typical anesthetics used in TBI models have the ability to pose as a significant confounding factor, given some of their neurotoxic and neuroprotective effects. The mice within this study were helmeted, with their heads free to move into a foam base after impact. The strategies implemented in this model strive to replicate, as precisely as possible, the environment with which humans are subjected to prior to being afflicted with the untoward effects of full-blown CTE. Some of the neurobehavioral changes seen in humans diagnosed with CTE could be replicated in mice with this novel model. In addition, a variety of neuropathological changes typically seen in CTE was demonstrated as well [37]. Further refinement of this new model will inevitably take place in the future, allowing researchers and clinicians to bridge the gap between the lab and clinical arena.

## Conclusions

Chronic traumatic encephalopathy is a serious neuropsychiatric condition, often overlooked because of the paucity of early dramatic symptoms and signs, as well as a lack of findings on routine radiographic imaging. However, mTBI can be a disabling condition, leading to both emotional and physical abnormalities later on in an athlete's life. Routine neuroradiographic imaging generally does not show any significant findings; however, newer MRI-based techniques, such as DTI, fMRI, and MRS, have shown promise for earlier detection. Clinical presentation of CTE typically occurs in three stages. Once the third stage is reached, full blown Parkinsonism if often encountered. Our understanding today is that CTE likely results from progressive neuronal loss, and that once the cascades are started, the symptoms of mTBI continue to progress. This further highlights the paramount importance of sport-specific preventive strategies and early detection in our athletes.
